# End of Life in Intensive Care Unit

**Published:** 2011-10-17

**Authors:** Giuseppe Servillo, Maria Vargas

**Affiliations:** Dipartimento di Scienze Chirurgiche, Anestesiologiche, Rianimatorie e dell’Emergenza - University of Naples “Federico II”

Intensive care unit (ICU) is a setting in which the most technologically and advanced treatments are used to manage severe and acute diseases. The aim of such life sustaining treatment is to preserve and restore the critical patient’s life quality [1]. Therefore, the nature of ICU is to give the best life care to discharge the patients previously admitted. Nevertheless, the ICU has become a common place to die:in the United States 22% of death occur in/or after admission in ICU [2]. In this contest the life sustaining care has become the End of Life care, showing the paradox of the treatments provided here. The medical actions, whose aim is life preservation, became procedures that improve the quality and comfort of death.

End-of-life care in ICU includes withholding and withdrawing life sustaining therapies and active euthanasia, the latter only in some European countries when allowed by law [3]. Withdrawal of therapies is defined as the removal of life sustaining treatments; withholding concerns the concept of no therapeutic escalation [4]. These two condition don’t differ from medical-legal point of view.

Euthanasia is defined in Belgium law as “action on the part of a third person intended to end the life of someone who has requested it” [5]. These special cares concern four types of patients [6]:

- Conscious patients, which are not at the end of life, such as patients with chronic and degenerative neurologic illnesses as amyotrophic lateral sclerosis.
- Unconscious patients, which are not at the end of life. E.g. patients in a vegetative state condition, etc.
- Conscious patients, at the end of life suffering (for example from terminal cancer) from end stage diseases.
- Unconscious patients, at the end of life affected by serious critical illnesses as ARDS, MOFS, septic shock, for whom aggressive treatments are ineffective too.

The problem between the first two categories of patients is their poor prognosis. In this situation all the medical actions don’t improve their quality of life but just prolonged it [7]. For the last two categories of patients, the organs failure is so wide that medical treatment unnecessarily prolongs their agony.

## Withholding or Withdrawal Treatments

In the ICU a large proportion of deaths are preceded by withholding or withdrawal treatments [8]. In a large international study including data from 282 ICU, 36.2% of hospital deaths occurred after decisions to forgo life-sustaining treatments [9]. Among these data, 54.6% patients received withholding decisions and 45.4% patients received withdrawing decisions [9]. Clinical condition underlying these choices are various and different. In the epidemiologic study by Angus, Human Immunodeficiency Virus (HIV) or Acquired Immunodeficiency Syndrome (AIDS), metastatic malignancy, cerebrovascular disease, acute myocardial infarction (AMI), and pneumonia represented 54.3% of all deaths after ICU admission [2]. The retrospective cohort study by Hall and Rocker, showed that sepsis and organ failure accounted for most of deaths in patients for whom life support was withdrawn, whereas sudden catastrophic events (e.g. hemorrhage, pulmonary embolism) were common in patients dying despite active support. Neurologic injury was more prevalent among patients from whom life support was withheld or withdrawn. [10]. These data suggest that end of life cares concern severe and acute illnesses with new onset or acute condition that worsen a chronic or persistent state.

## End of Life in Italy

In Italy the debate about end of life care is still in progress and it is widely influenced by political, cultural and religious traditions of this society. The catholic church has a clear position about this issue with the full opposition to the euthanasia and to the interruption of any treatment [11]. However many doctors often have a no definite position on the end of life care probably due to the lack of a clear legislation for withdrawing and withholding life supporting therapies. Although Italy ratified the Oviedo Convention, a major problem is that, as with many other countries, Italy does not have clear-cut laws related to end-of-life careyet, thus making very difficult or impossible to define whether a physician has theauthority to make decisions about end-of-life care regarding limiting or withholding support in terminally ill patients. In fact, as euthanasia and physician-assisted suicide areillegal in Italy, any policy dealing with withdrawal or withholding of therapies must first prove that these current laws have not been violated [12]. Recently, the drama of Piergiorgio Welby and Eluana Englaro have proved the real need of a law, which rules the living will and the end-of-life care of either conscious or unconscious patients. Despite the rejection of a court in Rome of Welby’s request to have a physician to switch off his life support machine, an anaesthesiologist of the Hospital of Cremona, Dr Riccio, administrated an intravenous cocktail of sedatives and disconnected the respirator. Dr Riccio justified his action quoting the Article 32 of the Italian Constitution that gives the patient the right to refuse medical treatment. The case of Mrs Englaro has been particularly ubiquitous media coverage [13]. For the first time in Italy after this case, the supreme court allowed withdrawal of tube feeding with the complete awareness that this act could lead to a patient’s death [14]. This decision was ground-breaking in this country, where catholic beliefs influence political discourse on this subject, and it was also the main cause of a new national law currently under discussion in the italian parliament [15]. This new law states the full opposition to futile therapy and for the first time in Italy introduces the advanced directives of treatment (Adt) defined as instructions, given by individuals, which will specify what medical treatments should be taken, for their health and their life, if they are no longer capable to make decisions [15]. In agreement with the ‘United Nation Convention on the right of persons with disabilities’ [16], this new act states that feeding and hydration are life-supporting therapies physiologically finalized to alleviate the suffering till the end of the life, and leaving them outside from the advanced directives. This statement agrees with the believes of the catholic church that doesn’t consider assisted nutrition and hydration (AHN) as a therapy and disagrees with the Italian society of intensive care (SIAARTI) that considers them, in critically ill patients, as a medical treatment that falls within the specific area of intensive care [17]. In this situation 61% of 22.219 Italian physicians, respondent to a questionnaire about End of Life decision, consider AHN to be a medical treatment, although 37% of them believe that the tube feeding is a life sustaining treatment [14]. About the decision to withheld or withdraw treatments, the physicians of 84 Italian ICU involved in GiViTi Project, affirm that these decisions was made in the 37.5% of 3168 terminally ill patients [18].

## End of Life in other Countries

In the April 2005, the french parliament voted a law called ‘patients’ right and the end of life’ (also termed as Leonetti’s law) after the lay press reported the withdrawal of mechanical ventilation in a case of locked-in syndrome [19]. This law now authorizes the stopping of life support when deemed futile, if the patients request it. However, it goes further, also allowing physicians to withdraw all ‘active treatments’, in the case of incompetent patients, if some precisely defined safeguards are respected: after seeking the advice of family members, with the decision being taken collectively with other physicians, and written in medical charts [20]. Administration of opiates according to the ‘double effect’standard is also allowed [20]. In passing this law, the French Parliament pursued essentially two objectives: to acknowledge a patient’s right to oppose unreasonable obstinacy; and to delineate good medical practices, both when a patient is conscious or unconscious, and whether or not they are at the end of life [21]. The main change it introduced is the possibility for physicians in France to withhold or even withdraw life support for unconscious patients [22].In Canada and other Anglo-American jurisdictions, a patient’s unequivocal right to refuse medical treatment is well established and is ethically justified by the principle of autonomy, according to which people have a right to self-governance, to act freely in accordance with a self-chosen plan. Control over the body has been taken to be central to the interpretation of autonomy [23]. In the United States, physicians remain the *de facto* arbiters for most aspects of medical care, including end-of-life interventions. All states allow patients to refuse medical treatments [24]. In the United States, the withholding and withdrawal of life support is legally justified primarily by the principles of informed consent and informed refusal, both of which have strong roots in the common law. The principles hold that treatment may not be initiated without the approval of patients or their surrogates excepting in emergency situations, and patients or surrogates may refuse any or all therapies [25]. In accord with this approach, federal guidelines require hospitals to ask all patients at the time of admission whether they would like to fill out an ‘advance directive,’ naming the person whom they would like to make decisions on their behalf if they should become incapable of making decisions for themselves [26].

## CONCLUSIONs

End of life cares are characterized by great variability in patient selection, treatments (withdrawn or withheld), type of palliative care and different national laws, which regulate them. Both religious, cultural, scientific and ethics reasons are responsible for these wide differences. Therefore, we hope that in a close futurenational barriers will be broken down, and the processes of end of life will be globally standardized, in order to ensure the dignity of the patients with the best and most clear care possible, beyond national borders and law.
